# Supplementary material for: Toxicity Studies of Cardiac-Targeting Peptide Reveal a Robust Safety Profile
Source: Pharmaceutics. 2024 Jan 4;16(1):73. doi: 10.3390/pharmaceutics16010073 (PMC10818749; doi:10.3390/pharmaceutics16010073)
Supplement: Supplementary file 1 [file pharmaceutics-16-00073-s001.zip › pharmaceutics-2759464-supplementary.pdf]

Toxicity Studies of Cardiac Targeting Peptide Reveal a Robust Safety Profile: Supplemental Material

Daniella Sahagun[1], BS, Jack Lopuszynski[1], BS; Kyle Feldman[2], BS, Nicholas Pogodzinski[3], BS; Maliha Zahid[1], MBBS, PhD

[1]Department of Cardiovascular Medicine, Mayo Clinic, Rochester, MN

[2]Clinical Virology Laboratory, Yale New Haven Hospital, New Haven, CT

[3]University of Pittsburgh, Pittsburgh, PA

Corresponding Author: Maliha Zahid
Guggenheim Gu9-01B
Mayo Clinic
200 First Street SW
Rochester, MN55905
Email: Zahid.maliha@mayo.edu

Funding: This work was supported by the Pitt Innovation Challenge, a program of the Clinical and Translational Science Institute at the University of Pittsburgh. DS, JL and MZ are supported by NIH grant R01HL153407, both awarded to MZ.

Conflict of Interest: MZ holds a patent on use of cardiac targeting peptide for cardiac delivery along with Dr. Paul Robbins and University of Pittsburgh, PA.

Supplemental Methods S1

Continued on next page.

eurofins | DISCOVERY

Release Date: 29 Nov 2022

Supplemental Material S1: Full and detailed methods of the Eurofins Ion Channel Cardiac Profiler Panel as performed and reported by Eurofins.

REPORT

Study Period | 07 Nov 2022 - 28 Nov 2022

STUDY OBJECTIVE:

Client Name: Mayo Clinic
Compound Names: CTP
Tested in IonChannelProfiler™ CardiacProfiler Panel

STUDY INFORMATION:

Study code#: US034-0018974

Eurofins Discovery

6 Research Park Drive
St Charles MO 63304
USA

T | +1 844 522 7787
F | +1 636 362 7131
www.eurofins.com

eurofins | DISCOVERY

COMPOUND INFORMATION

Test compounds

Eurofins Compound I.D.	Client Compound I.D.	Client Reference I.D.	MW	MW + Salt	Weight received (mg)	Received condition
US034-0018974-2	CTP	1		1432.00	3	Dry Powder

Reference compounds

In each experiment and if applicable, the respective reference compounds were tested concurrently with the test compounds, and the data were compared with historical values determined at Eurofins. The experiment was accepted in accordance with Eurofins Standard Operating Procedure on assay validation.

SUMMARY

Ion Channels tested

Panel Part Number: CPROFullQB2(DR)

Voltage-Gated Sodium: HEK-Nav1.5 (Peak), HEK-Nav1.5 (Late, Antagonist)
Voltage-Gated Potassium: HEK-Kv4.3/KChIP2, CHO-hERG, CHO-KCNQ1/minK
Voltage-Gated Calcium: HEK-Cav1.2
Inward-Rectifying Voltage-Gated Potassium: HEK-Kir2.1

Study objective

Electrophysiological assays conducted to profile two (2) compounds for activities on the ion channel targets specified above using the Qube electrophysiological platform.

Measurements

Methods employed in this study have been developed and validated with reliability and reproducibility. Assays were performed under conditions described in the accompanying "Materials and Methods" section of this report.

Where presented, IC_{50} values were determined by a non-linear, least squares regression analysis. Reference standards were run as an integral part of each assay to ensure the validity of the results obtained.

Significant results

Results showing an inhibition greater than 50% are considered to represent significant effects of test compounds and listed in the following tables with individual calculation results or calculable IC_{50}.

DATA CALCULATION AND ANALYSIS

CardiacProfiler Qube Panel

All compounds were tested in the presence of 0.1% Pluronic F-68 Non-Ionic Surfactant for all assays.

CYL8004QB2DR hNav1.5 Sodium Channel Assay – Qube APC
The parameters measured were difference between the peak inward current on stepping to -15mV (i.e. peak of the current) and the leak current. All data were filtered for seal quality, seal drop, and current amplitude. The peak current amplitude was calculated before and after compound addition and the amount of block was assessed by dividing the Test compound current amplitude by the Control current amplitude. Control is the mean Nav1.5 current amplitude collected 10 seconds at the end of the vehicle control; Test Compound is the mean Nav1.5 current amplitude collected 10 seconds at the end of test concentration application for each concentration.

CYL8069QB2DR hKv4.3/hKChIP2 Potassium Channel Assay – Qube APC
The parameters measured were the maximum outward current evoked on stepping to 40mV from holding potential of -80mV. All data were filtered for seal quality, seal drop, and current amplitude. The peak current amplitude was calculated before and after compound addition and the amount of block was assessed by dividing the Test compound current amplitude by the Control current amplitude. Control data is the mean Kv4.3/KChIP2 current amplitude collected 10 seconds at the end of the vehicle control period; Test compound data is the mean Kv4.3/KChIP2 current amplitude collected 10 seconds at the end of test concentration application for each concentration.

CYL8051QB2DR hCav1.2 (L-type) Calcium Channel Assay – Qube APC
The parameters measured were the maximum inward current elicited on stepping from -100mV to +20mV for 200ms. All data were filtered for seal quality, seal drop, and current amplitude. The peak current amplitude was calculated before and after compound addition and the amount of block was assessed by dividing the Test compound current amplitude by the Control current amplitude. Control data is the mean Cav1.2 current amplitude collected at the end of the vehicle control; Test compound data is the mean Cav1.2 current amplitude collected at the end of test concentration application for each concentration.

CYL7004QB2DR hNav1.5 Late Current Sodium Channel Assay – Qube APC
The parameters measured were the ramp current charge (AUC) evoked on ramping back to -80mV from 40mV test pulse in the presence of 50nM ATXII. All data were filtered for seal quality, seal drop, and current. The peak and ramp current amplitude was calculated before and after compound addition and the amount of current was assessed by dividing the Test compound current by the Control current. Control data is the mean hNav1.5 late current collected 15 seconds at the end of 50nM ATXII application (50nM ATXII control); Test compound data is the mean ramp hNav1.5 current collected 15 seconds at the end of test concentration application for each concentration.

CYL8038QB2DR hERG Potassium Channel Assay - Qube APC
The parameters measured were the maximum tail current evoked ramping back to -80mV from the test pulse of 40mV. All data were filtered for seal quality, seal drop, and current amplitude. The peak current amplitude was calculated before and after compound addition and the amount of block was assessed by dividing the Test compound current amplitude by the Control current amplitude. Control data is the mean hERG current amplitude collected 15 seconds at the end of the vehicle control; Test compound data is the mean hERG current amplitude collected 15 seconds at the end of test concentration application for each concentration.

CYL8007QB2DR hKCNQ1/hminK Potassium Channel Assay – Qube APC
The parameters measured were the maximum outward current evoked on stepping to +60mV from a holding potential of -80mV. All data were filtered for seal quality, seal drop, and current amplitude. The peak current amplitude was calculated before and after compound addition and the amount of block was assessed by dividing the Test compound current amplitude by the Control current amplitude. Control data is the mean hKCNQ1/hminK current amplitude collected 30 seconds at the end of vehicle control period; Test compound data is the mean hKCNQ1/hminK current amplitude collected 30 seconds at the end of test concentration application for each concentration.

CYL8032QB2DR hKir2.1 Potassium Channel Assay – Qube APC

The parameters measured were the maximum inward current elicited on stepping to -120mV for 500ms from a holding potential of -30 mV. All data were filtered for seal quality, seal drop, and current amplitude. The peak current amplitude was calculated before and after compound addition. Residual non-Kir2.1 current was eliminated via normalization to residual current after application of 100uM Barium Chloride. The amount of Test compound effect was then assessed by dividing the Test compound current amplitude by the Control current amplitude. Control data is the mean Kir2.1 current amplitude collected 40 seconds at the end of the vehicle control; Test compound data is the mean Kir2.1 current amplitude collected 30 seconds at the end of test concentration application for each concentration.

MATERIALS AND METHODS

CardiacProfiler Qube Protocols

CYL8004QB2DR hNav1.5 Sodium Channel Assay – Qube APC
Onset and steady state block of peak Nav1.5 current is measured using a pulse pattern, repeated every 5 sec, consisting of a hyperpolarizing pulse to -120mV for a 200ms duration, depolarization to -15mV amplitude for a 40ms duration, followed by step to 40mV for 200ms and finally a 100ms ramp (1.2 V/s) to a holding potential of -80mV. Peak current is measured during the step to -15mV.

CYL8069QB2DR hKv4.3/hKChIP2 Potassium Channel Assay – Qube APC
After whole cell configuration is achieved, the cells are held at -80mV. Onset and steady state block of hKv4.3 current is measured using a pulse pattern from -80mV to 40mV amplitude for a 110ms duration, and finally a 100ms ramp (1.2 V/s) to -80mV. This paradigm is delivered once every 5s to monitor the current amplitude.

CYL8051QB2DR hCav1.2 (L-type) Calcium Channel Assay – Qube APC
After whole cell configuration is achieved, the cells are held at -90mV. Cav1.2 currents are evoked by a 50ms pulse to -100mV followed by a 200ms pulse to +20mV before returning to the holding potential of -90mV. This paradigm is delivered three times every 60s to monitor the current amplitude.

CYL7004QB2DR hNav1.5 Late Current Sodium Channel Assay – Qube APC
Onset and steady state block of Late Nav1.5 current is measured using a pulse pattern, repeated every 5 sec, consisting of a hyperpolarizing pulse to -120mV for a 200ms duration, depolarization to -15mV amplitude for a 40ms duration, followed by step to 40mV for 200ms and finally a 100ms ramp (1.2 V/s) to a holding potential of -80mV. Late current is measured as charge current elicited during the ramp with 50nM ATXII.

CYL8038QB2DR hERG Potassium Channel Assay - Qube APC
After whole cell configuration is achieved, the cells are held at -80mV. Cells are held at this voltage for 50ms to measure the leak current, which is subtracted from the tail current on-line. The cells are depolarized to +40mV for 500ms and then to -80 mV over a 100ms ramp to elicit the hERG tail current. This paradigm is delivered once every 8s to monitor the current amplitude.

CYL8007QB2DR hKCNQ1/hminK Potassium Channel Assay – Qube APC
After whole cell configuration is achieved, the cells are held at -80mV. KCNQ1/minK currents are evoked by a 1000ms pulse from -80mV to 60mV followed by a ramp from 60mV to -80mV over 115ms with the outward peak currents measured upon depolarization of the cell membrane. This paradigm is delivered once every 15s to monitor the current amplitude.

CYL8032QB2DR hKir2.1 Potassium Channel Assay – Qube APC
After whole cell configuration is achieved, the cells are held at -30mV. Kir2.1 currents are evoked by a single 500ms pulse to -120mV before returning to the holding potential of -30mV. This paradigm is delivered once every 20s to monitor the current amplitude.

STORAGE AND RETENTION OF RECORDS

Documents generated during the performance of the study will be archived by Eurofins Discovery for a period of time after study completion (this period of time is dependent on each individual site policy). The access to the archives is restricted to authorized employees only.

Supplemental Methods S2

Continued on next page.

Supplemental Methods S2: Full and detailed methods of the Eurofins SAFETYscan E/IC50 ELECT Service, including principles and methods, as performed and reported by Eurofins.

Study Report

Requester(s): Maliha Zahid

Company: Mayo Clinic

Study Date: 06/21/2023

Report Date: 06/21/2023

Quote ID: US073-0027327-Q

Order ID: US073-0027327-O

Service: SAFETYscan E/IC50 ELECT

Number of Compounds Tested: 1

Number of Assays Tested: 78

Associate Director of Screening: Mirza Jahic

Phone: (858) 224-6930

www.discoverx.com

Eurofins DiscoverX Corporation

11180 Roselle Street, Suite D

San Diego, CA 92121

Technology Principle

cAMP Secondary Messenger Assay

DiscoverX has developed a panel of cell lines stably expressing non-tagged GPCRs that endogenously signal through cAMP. Hit Hunter® cAMP assays monitor the activation of a GPCR via Gi and Gs secondary messenger signaling in a homogenous, non-imaging assay format using a technology developed by DiscoverX called Enzyme Fragment Complementation (EFC) with β-galactosidase (β-gal) a the functional endpoint.

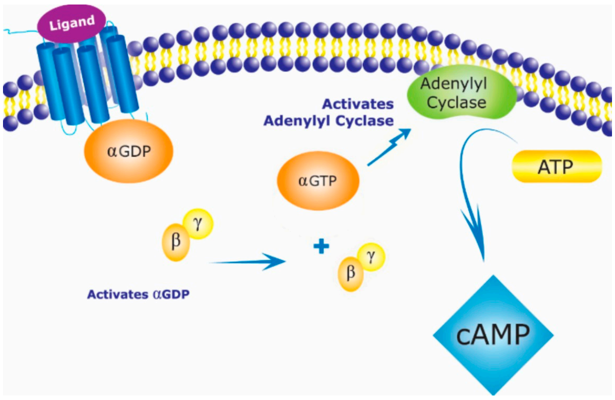

The enzyme is split into two complementary portions: Enzyme Acceptor (EA) and Enzyme Donor (ED). In the assay, exogenously introduced ED fused to cAMP (ED-cAMP) competes with endogenously generated cAMP for binding to an anti-cAMP-specific antibody. Active β-gal is formed by complementation of exogenous EA to any unbound ED-cAMP. Active enzyme can then convert a chemiluminescent substrate, generating an output signal detectable on a standard microplate reader.

Calcium Secondary Messenger Assay

The Calcium No Wash^{PLUS} assay monitors GPCR activity via Gq secondary messenger signaling in a live cell, non-imaging assay format.

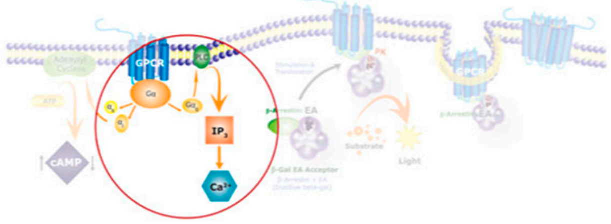

Calcium mobilization in PathHunter® cell lines or other cell lines stably expressing Gq-coupled GPCRs is monitored using a calcium-sensitive dye that is loaded into cells. GPCR activation by a compound results in the release of calcium from intracellular stores and an increase in dye fluorescence that is measured in real-time.

Nuclear Hormone Receptor Assay

PathHunter® NHR Protein Interaction (NHR Pro) and Nuclear Translocation (NHR NT) assays monitor the activation of a nuclear hormone receptor in a homogenous, non-imaging assay format using a technology developed by DiscoverX called Enzyme Fragment Complementation (EFC).

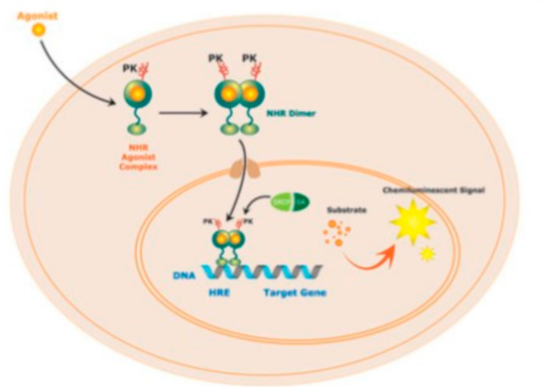

The NHR Pro assay is based on detection of protein-protein interactions between an activated, full length NHR protein and a nuclear fusion protein containing Steroid Receptor Co-activator Peptide (SRCP) domains with one or more canonical LXXLL interaction motifs.

The NHR is tagged with the ProLink™ (PK) component of our EFC assay system, and the SRCP domain is fused to the Enzyme Acceptor component (EA) expressed in the nucleus. When bound by ligand, the NHR will migrate to the nucleus and recruit the SRCP domain, whereby complementation occurs, generating a unit of active β-galactosidase (β-gal) and production of chemiluminescent signal upon the addition of PathHunter detection reagents.

Benefits associated with this approach include reduced compound incubation times, direct measurement of the NHR target, use of full-length human NHR sequences, and the ability to select novel compound classes based on disruption of protein-protein interactions.

The NHR NT assay monitors movement of an NHR between the cytoplasmic and nuclear compartments. The receptor is tagged with the ProLabel™ (PL) component of our EFC assay system, and EA is fused to a nuclear location sequence that restricts the expression of EA to the nucleus. Migration of the NHR to the nucleus results

in complementation with EA generating a unit of active β-gal and production of a chemiluminescent signal upon the addition of PathHunter detection reagents.

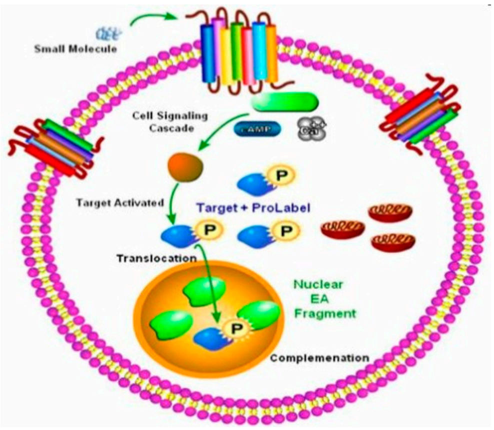

KINOMEscan® Assay

The KINOME*scan* screening platform employs a novel and proprietary active site-directed competition binding assay to quantitatively measure interactions between test compounds and the kinases. This robust and reliable assay technology affords investigators the ability to extensively annotate compounds with accurate, precise and reproducible data. KINOME*scan* assays do not require ATP and thereby report true thermodynamic interaction affinities.

Compounds that bind the kinase active site and directly (sterically) or indirectly (allosterically) prevent kinase binding to the immobilized ligand, will reduce the amount of kinase captured on the solid support (A and B). Conversely, test molecules that do not bind the kinase have no effect on the amount of kinase captured on the solid support (C). Screening "hits" are identified by measuring the amount of kinase captured in test versus control samples by using a quantitative, precise and ultra-sensitive qPCR method that detects the associated DNA label (D). In a similar manner, dissociation constants (Kds) for test compound-kinase interactions are calculated by measuring the amount of kinase captured on the solid support as a function of the test compound concentration.

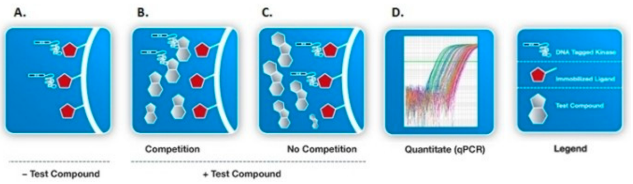

Neurotransmitter Transporter Uptake Assay

The Neurotransmitter Transporter Uptake Assay Kit from Molecular Devices was used and it provides a homogeneous fluorescence based assay for the detection of dopamine, norepinephrine or serotonin transporter activity in cells expressing these transporters. The kit employs a fluorescent substrate that mimics the biogenic amine neurotransmitters that are taken into the cell through these specific transporters, resulting in increased intracellular fluorescence intensity. This homogeneous, fluorescent assay can be run in either a kinetic or an endpoint mode.

Potassium Assay

The FLIPR Potassium Assay Kit from Molecular Devices is used. It exploits the permeability of thallium ions (Tl+) through both voltage- and ligand-gated potassium (K+) channels. A novel, highly-sensitive Tl+ indicator dye is utilized which produces a bright fluorescent signal upon the binding to Tl+ conducted through potassium channels. The intensity of the Tl+ signal is proportional to the number of potassium channels in the open state; therefore it provides a functional indication of the potassium channel activities. In addition, a proprietary masking dye is included to further reduce background fluorescence for improved signal/noise ratio.

Membrane Potential Assay

The FLIPR® Membrane Potential Assay Kit is used which utilizes a proprietary fluorescent indicator dye in combination with a quencher to reflect real-time membrane potential changes associated with ion channel activation and ion transporter proteins. Unlike traditional dyes such as DiBAC, the FLIPR Membrane Potential Assay Kit detects bidirectional ion fluxes so both variable and control conditions can be monitored within a single experiment.

Calcium Assay

The DiscoveRx Calcium NWPLUS Assay Kit was used for detection of changes in intracellular calcium. Cells expressing a receptor of interest that signals through calcium are pre-loaded with a calcium-sensitive dye and then treated with compound. Upon stimulation, the receptor signals release of intracellular calcium, which results in an increase of dye fluorescence. This kit provides a homogenous assay format for detection of calcium mobilization. Signal is measured on a fluorescent plate reader equipped with fluidic handling capable of detecting rapid changes in fluorescence upon compound stimulation.

Enzymatic Assays

Enzymatic assays determine enzymatic activity by measuring either the consumption of substrate or production of product over time. Different detection methods are used in each enzymatic assay to measure the concentrations of substrates and products, including spectrophotometric, fluorometric, and luminescent readouts.

Profile Overview

DiscoverX was contracted by Maliha Zahid at Mayo Clinic to profile 1 compound(s) with the SAFETYscan E/IC50 ELECT service. Details are given below:

Product: SAFETYscan E/IC50 ELECT

Assays: 78

Number of Compounds: 1

Data Points: 780

Targets:

ADORA2A-Calcium Flux-Agonist
ADORA2A-Calcium Flux-Antagonist
ADRA1A-Calcium Flux-Agonist
ADRA1A-Calcium Flux-Antagonist
ADRA2A-cAMP-Agonist
ADRA2A-cAMP-Antagonist
ADRB1-cAMP-Agonist
ADRB1-cAMP-Antagonist
ADRB2-cAMP-Agonist
ADRB2-cAMP-Antagonist
AVPR1A-Calcium Flux-Agonist
AVPR1A-Calcium Flux-Antagonist
CCKAR-Calcium Flux-Agonist
CCKAR-Calcium Flux-Antagonist
CHRM1-Calcium Flux-Agonist
CHRM1-Calcium Flux-Antagonist
CHRM2-cAMP-Agonist
CHRM2-cAMP-Antagonist
CHRM3-Calcium Flux-Agonist
CHRM3-Calcium Flux-Antagonist
CNR1-cAMP-Agonist
CNR1-cAMP-Antagonist
CNR2-cAMP-Agonist
CNR2-cAMP-Antagonist
DRD1-cAMP-Agonist
DRD1-cAMP-Antagonist
DRD2S-cAMP-Agonist
DRD2S-cAMP-Antagonist
EDNRA-Calcium Flux-Agonist
EDNRA-Calcium Flux-Antagonist
HRH1-Calcium Flux-Agonist
HRH1-Calcium Flux-Antagonist
HRH2-cAMP-Agonist
HRH2-cAMP-Antagonist
HTR1A-cAMP-Agonist
HTR1A-cAMP-Antagonist
HTR1B-cAMP-Agonist
HTR1B-cAMP-Antagonist
HTR2A-Calcium Flux-Agonist
HTR2A-Calcium Flux-Antagonist
HTR2B-Calcium Flux-Agonist
HTR2B-Calcium Flux-Antagonist
OPRD1-cAMP-Agonist
OPRD1-cAMP-Antagonist
OPRK1-cAMP-Agonist
OPRK1-cAMP-Antagonist
OPRM1-cAMP-Agonist
OPRM1-cAMP-Antagonist
CAV1.2-Ion Channel-Blocker
GABAA-Ion Channel-Blocker
GABAA-Ion Channel-Opener
hERG-Ion Channel-Blocker
HTR3A-Ion Channel-Blocker
HTR3A-Ion Channel-Opener
KvLQT1/minK-Ion Channel-Blocker
KvLQT1/minK-Ion Channel-Opener
nAChR(a4/b2)-Ion Channel-Blocker
nAChR(a4/b2)-Ion Channel-Opener
NAV1.5-Ion Channel-Blocker
NMDAR (1A/2B)-Ion Channel-Blocker
NMDAR (1A/2B)-Ion Channel-Opener
INSR-Binding-Inhibitor
LCK-Binding-Inhibitor
ROCK1-Binding-Inhibitor
VEGFR2-Binding-Inhibitor
AR-NHR Nuclear Translocation-Agonist
AR-NHR Nuclear Translocation-Antagonist
GR-NHR Protein Interaction-Agonist
GR-NHR Protein Interaction-Antagonist
AChE-Enzymatic-Inhibitor
COX1-Enzymatic-Inhibitor
COX2-Enzymatic-Inhibitor
MAOA-Enzymatic-Inhibitor
PDE3A-Enzymatic-Inhibitor
PDE4D2-Enzymatic-Inhibitor
DAT-Transporter-Blocker
NET-Transporter-Blocker
SERT-Transporter-Blocker

Assay Design: GPCR cAMP Modulation

Cell Handling

1. cAMP Hunter cell lines were expanded from freezer stocks according to standard procedures.
2. Cells were seeded into white-walled, 384-well microplates and incubated at 37°C for the appropriate time prior to testing.
3. cAMP modulation was determined using the DiscoverX HitHunter cAMP XS+ assay.

Gs Agonist Format

1. For agonist determination, cells were incubated with sample to induce response.
2. Media was aspirated from cells and replaced with 15 μL 2:1 HBSS/10mM Hepes : cAMP XS+ Ab reagent.
3. Intermediate dilution of sample stocks was performed to generate 4X sample in assay buffer.
4. 5 μL of 4X sample was added to cells and incubated at 37°C or room temperature for 30 or 60 minutes.

Gi Agonist Format

1. For agonist determination, cells were incubated with sample in the presence of EC_{80} forskolin to induce response.
2. Media was aspirated from cells and replaced with 15 μL 2:1 HBSS/10mM Hepes : cAMP XS+ Ab reagent.
3. Intermediate dilution of sample stocks was performed to generate 4X sample in assay buffer containing 4X EC_{80} forskolin.
4. 5 μL of 4X sample was added to cells and incubated at 37°C or room temperature for 30 or 60 minutes.

Antagonist Format

1. For antagonist determination, cells were pre-incubated with sample followed by agonist challenge at the EC_{80} concentration.
2. Media was aspirated from cells and replaced with 10 μL 1:1 HBSS/Hepes : cAMP XS+ Ab reagent.
3. 5 μL of 4X compound was added to the cells and incubated at 37°C or room temperature for 30 minutes.
4. 5 μL of 4X EC_{80} agonist was added to cells and incubated at 37°C or room temperature for 30 or 60 minutes. For Gi coupled GPCRs, EC_{80} forskolin was included.

Signal Detection

1. After appropriate compound incubation, assay signal was generated through incubation with 20 μL cAMP XS+ ED/CL lysis cocktail for one hour followed by incubation with 20 μL cAMP XS+ EA reagent for three hours at room temperature.
2. Microplates were read following signal generation with a PerkinElmer Envision™ instrument for chemiluminescent signal detection.

Data Analysis

1. Compound activity was analyzed using CBIS data analysis suite (ChemInnovation, CA).
2. For Gs agonist mode assays, percentage activity was calculated using the following formula:
   % Activity =100% x (mean RLU of test sample - mean RLU of vehicle control) / (mean RLU of MAX control - mean RLU of vehicle control).
3. For Gs antagonist mode assays, percentage inhibition was calculated using the following formula:
   % Inhibition =100% x (1 - (mean RLU of test sample - mean RLU of vehicle control) / (mean RLU of EC_{80} control - mean RLU of vehicle control)).
4. For Gi agonist mode assays, percentage activity was calculated using the following formula:
   % Activity = 100% x (1 - (mean RLU of test sample - mean RLU of MAX control) / (mean RLU of vehicle control - mean RLU of MAX control)).
5. For Gi antagonist or negative allosteric mode assays, percentage inhibition was calculated using the following formula:
   % Inhibition = 100% x (mean RLU of test sample - mean RLU of EC_{80} control) / (mean RLU of forskolin positive control - mean RLU of EC_{80} control).

For Primary screens, percent response was capped at 0% or 100% where calculated percent response returned a negative value or a value greater than 100, respectively.

eurofins | DISCOVERY

Assay Design: Calcium Mobilization

Cell Handling

1. Cell lines were expanded from freezer stocks according to standard procedures.
2. Cells were seeded into black-walled, clear-bottom, Poly-D-lysine coated 384-well microplates and incubated at 37°C for the appropriate time prior to testing.

Dye Loading

1. Assays were performed in 1X Dye Loading Buffer consisting of 1X Dye (DiscoverX, Calcium No Wash^{PLUS} kit, Catalog No. 90-0091), 1X Additive A and 2.5 mM Probenecid in HBSS / 20 mM Hepes. Probenecid was prepared fresh.
2. Cells were loaded with dye prior to testing. Media was aspirated from cells and replaced with 25 μL Dye Loading Buffer.
3. Cells were incubated for 45 minutes at 37°C and then 20 minutes at room temperature.

Agonist Format

1. For agonist determination, cells were incubated with sample to induce response.
2. After dye loading, cells were removed from the incubator and 25 μL of 2X compound in HBSS / 20 mM Hepes was added using a FLIPR Tetra (MDS).
3. Compound agonist activity was measured on a FLIPR Tetra. Calcium mobilization was monitored for 2 minutes with a 5 second baseline read.

Antagonist Format

1. For antagonist determination, cells were pre-incubated with sample followed by agonist challenge at the EC_{80} concentration.
2. After dye loading, cells were removed from the incubator and 25 μL 2X sample was added. Cells were incubated for 30 minutes at room temperature in the dark to equilibrate plate temperature.
3. After incubation, antagonist determination was initiated with addition of 25 μL 1X compound with 3X EC_{80} agonist using FLIPR
4. Compound antagonist activity was measured on a FLIPR Tetra (MDS). Calcium mobilization was monitored for 2 minutes with a 5 second baseline read.

Data Analysis

1. FLIPR read - Area under the curve was calculated for the entire two minute read.
2. Compound activity was analyzed using CBIS data analysis suite (ChemInnovation, CA).
3. For agonist mode assays, percentage activity was calculated using the following formula:
   % Activity =100% x (mean RFU of test sample - mean RFU of vehicle control) / (mean MAX RFU control ligand - mean RFU of vehicle control).
4. For antagonist mode assays, percentage inhibition was calculated using the following formula:
   % Inhibition =100% x (1 - (mean RFU of test sample - mean RFU of vehicle control) / (mean RFU of EC_{80} control - mean RFU of vehicle control)).

For Primary screens, percent response was capped at 0% or 100% where calculated percent response returned a negative value or a value greater than 100, respectively.

Assay Design: Nuclear Hormone Receptor

Cell Handling

1. PathHunter NHR cell lines were expanded from freezer stocks according to standard procedures.
2. Cells were seeded into white-walled, 384-well microplates and incubated at 37°C for the appropriate time prior to testing. Assay media contained charcoal-dextran filtered serum to reduce the level of hormones present.

Agonist Format

1. For agonist determination, cells were incubated with sample to induce response.
2. Intermediate dilution of sample stocks was performed to generate 5X sample in assay buffer.
3. 5 μL of 5X sample was added to cells and incubated at 37°C or room temperature for 3-16 hours.

Antagonist Format

1. For antagonist determination, cells were pre-incubated with antagonist followed by agonist challenge at the EC_{80} concentration.
2. Intermediate dilution of sample stocks was performed to generate 5X sample in assay buffer.
3. 5 μL of 5X sample was added to cells and incubated at 37°C or room temperature for 60 minutes. Vehicle concentration was 1%.
4. 5 μL of 6X EC_{80} agonist in assay buffer was added to the cells and incubated at 37°C or room temperature for 3-16 hours.

Signal Detection

1. Assay signal was generated through a single addition of 12.5 or 15 μL (50% v/v) of PathHunter Detection reagent cocktail, followed by a one hour incubation at room temperature.
2. Microplates were read following signal generation with a PerkinElmer Envision™ instrument for chemiluminescent signal detection.

Data Analysis

1. Compound activity was analyzed using CBIS data analysis suite (ChemInnovation, CA).
2. For agonist mode assays, percentage activity was calculated using the following formula:
   % Activity =100% x (mean RLU of test sample - mean RLU of vehicle control) / (mean MAX control ligand - mean RLU of vehicle control).
3. For antagonist mode assays, percentage inhibition was calculated using the following formula:
   % Inhibition =100% x (1 - (mean RLU of test sample - mean RLU of vehicle control) / (mean RLU of EC_{80} control - mean RLU of vehicle control)).
4. Note that for select assays, the ligand response produces an decrease in receptor activity (inverse agonist with a constitutively active target). For those assays inverse agonist activity was calculated using the following formula:
   % Inverse Agonist Activity =100% x ((mean RLU of vehicle control - mean RLU of test sample) / (mean RLU of vehicle control - mean RLU of MAX control)).

For Primary screens, percent response was capped at 0% or 100% where calculated percent response returned a negative value or a value greater than 100, respectively.

eurofins | DISCOVERY

Assay Design: KINOME*scan* Binding Assays

Protein Expression

For most assays, kinase-tagged T7 phage strains were grown in parallel in 24-well blocks in an *E. coli* host derived from the BL21 strain. *E. coli* were grown to log-phase and infected with T7 phage from a frozen stock (multiplicity of infection = 0.4) and incubated with shaking at 32˚C until lysis (90-150 minutes). The lysates were centrifuged (6,000 x g) and filtered (0.2 μm) to remove cell debris. The remaining kinases were produced in HEK-293 cells and subsequently tagged with DNA for qPCR detection.

Capture Ligand Production

Streptavidin-coated magnetic beads were treated with biotinylated small molecule ligands for 30 minutes at room temperature to generate affinity resins for kinase assays. The liganded beads were blocked with excess biotin and washed with blocking buffer (SeaBlock (Pierce), 1% BSA, 0.05% Tween 20, 1 mM DTT) to remove unbound ligand and to reduce non-specific phage binding.

Binding Reaction Assembly

Binding reactions were assembled by combining kinases, liganded affinity beads, and test compounds in 1X binding buffer (20% SeaBlock, 0.17X PBS, 0.05% Tween 20, 6 mM DTT). All reactions were performed in polypropylene 384-well plates in a final volume of 0.02 mL. The assay plates were incubated at room temperature with shaking for 1 hour and the affinity beads were washed with wash buffer (1x PBS, 0.05% Tween 20). The beads were then re-suspended in elution buffer (1x PBS, 0.05% Tween 20, 0.5 μM non-biotinylated affinity ligand) and incubated at room temperature with shaking for 30 minutes. The kinase concentration in the eluates was measured by qPCR.

Signal Detection

The kinase concentration in the eluates was measured by qPCR. qPCR reactions were assembled by adding 2.5 μL of kinase eluate to 7.5 μL of qPCR master mix containing 0.15 μM amplicon primers and 0.15 μM amplicon probe. The qPCR protocol consisted of a 10 minute hot start at 95˚C, followed by 35 cycles of 95˚C for 15 seconds, 60˚C for 1 minute.

Data Analysis

Percent Response Calculation

$$\frac{\text{test compound signal} - \text{positive control signal}}{\text{negative compound signal} - \text{positive control signal}} \times 100$$

Test compound = compound submitted by Customer

Negative control = DMSO (100%Ctrl)

Positive control = control compound (0%Ctrl)

Percent of Control was converted to Percent Response using formula: Percent Response = (100 - Percent Control).

For Primary screens, percent response was capped at 0% or 100% where calculated percent response returned a negative value or a value greater than 100, respectively.

Binding Constants (Kds)

Binding constants (Kds) were calculated with a standard dose-response curve using the Hill equation:

$$\text{Response} = \text{Background} + \frac{\text{Signal} - \text{Background}}{1 + \left(\text{Kd}^{\text{Hill Slope}} / \text{Dose}^{\text{Hill Slope}}\right)}$$

The Hill Slope was set to -1.

Curves were fitted using a non-linear least square fit with the Levenberg-Marquardt algorithm.

Assay Design: Ion Channel Assays

Cell Handling

1. Cell lines were expanded from freezer stocks according to standard procedures.
2. Cells were seeded into black-walled, clear-bottom, Poly-D-lysine coated 384-well microplates and incubated at 37°C for the appropriate time prior to testing.

Dye Loading

1. Assays were performed in 1X Dye Loading Buffer consisting of 1X Dye, and 2.5 mM Probenecid when applicable. Probenecid was prepared fresh.
2. Cells were loaded with dye prior to testing.
3. Cells were incubated for 30-60 minutes at 37°C.

Agonist/Opener Format

1. For agonist determination, cells were incubated with sample to induce response.
2. Intermediate dilution of sample stocks was performed to generate 2 - 5X sample in assay buffer.
3. 10-25 μL of 2 - 5X sample was added to cells and incubated at 37°C or room temperature for 30 minutes.

Antagonist/Blocker Format

1. For antagonist determination, cells were pre-incubated with sample.
2. Intermediate dilution of sample stocks was performed to generate 2 - 5X sample in assay buffer.
3. After dye loading, cells were removed from the incubator and 10 - 25 μL 2 - 5X sample was added to cells in the presence of EC_{80} agonist when appropriate. Cells were incubated for 30 minutes at room temperature in the dark to equilibrate plate temperature.

Signal Detection

1. Compound activity was measured on a FLIPR Tetra (MDS).

Data Analysis

1. Compound activity was analyzed using CBIS data analysis suite (ChemInnovation, CA).
2. For agonist mode assays, percentage activity was calculated using the following formula:
   % Activity =100% x (mean RLU of test sample - mean RLU of vehicle control) / (mean MAX control ligand - mean RLU of vehicle control).
3. For antagonist percentage inhibition was calculated using the following formula:
   % Inhibition =100% x (1 - (mean RLU of test sample - mean RLU of vehicle control) / (mean RLU of EC_{80} control - mean RLU of vehicle control)).

For Primary screens, percent response was capped at 0% or 100% where calculated percent response returned a negative value or a value greater than 100, respectively.

Assay Design: Transporter Assays

Cell Handling

1. Cell lines were expanded from freezer stocks according to standard procedures.
2. Cells were seeded into black-walled, clear-bottom, Poly-D-lysine coated 384-well microplates and incubated at 37°C for the appropriate time prior to testing.

Blocker/Antagonist Format

1. After cell plating and incubation, media was removed and 25 μL of 1X compound in 1X HBSS/0.1% BSA was added.
2. Compounds were incubated with cells at 37°C for 30 minutes.

Dye Loading

1. Assays were performed in 1X Dye Loading Buffer consisting of 1X Dye, 1X HBSS / 20 mM Hepes.
2. After compound incubation, 25 μL of 1X dye was added to wells.
3. Cells were incubated for 30-60 minutes at 37°C.

Signal Detection

1. After dye incubation, microplates were transferred to a PerkinElmer Envision™ instrument for fluorescence signal detection.

Data Analysis

1. Compound activity was analyzed using CBIS data analysis suite (ChemInnovation, CA).
2. For blocker mode assays, percentage inhibition was calculated using the following formula:
   % Inhibition =100% x (1 - (mean RLU of test sample - mean RLU of vehicle control) / (mean RLU of positive control - mean RLU of vehicle control)).

For Primary screens, percent response was capped at 0% or 100% where calculated percent response returned a negative value or a value greater than 100, respectively.

Assay Design: Enzymatic Assays

Enzyme Preparations

Enzyme preparations were sourced from various vendors-AChE (R&D Systems), COX1 and COX2 (BPS Bioscience), MAOA (Sigma), PDE3A and PDE4D2 (Signal Chem).

Enzyme Activity Assays

1. Enzymatic assays determine the enzymatic activity by measuring either the consumption of substrate or production of product over time. Different detection methods were used in each enzymatic assay to measure the concentrations of substrates and products.

2. AChE: Enzyme and test compound were preincubated for 15 minutes at room temp before substrate addition. Acetylthiocholine and DTNB were added and incubated at room temperature for 30 minutes. Signal was detected by measuring absorbance at 405 nm.

3. COX1 & COX2: Enzyme stocks were diluted in Assay Buffer (40 mM Tris-HCl, 1X PBS, 0.5 mM Phenol, 0.01% Tween-20 + 100 nM Hematin) and allowed to equilibrate with compounds at room temperature for 30 minutes (binding incubation). Arachidonic acid (1.7 μM) and Ampliflu Red (2.5 μM) were prepared and dispensed into a reaction plate. Plates were read immediately on a fluorimeter with the emission detection at 590 nm and excitation wavelength 544 nm.

4. MAOA: Enzyme and test compound were preincubated for 15 minutes at 37°C before substrate addition. The reaction was initiated by addition of kynuramine and incubated at 37°C for 30 minutes. The reaction was terminated by addition of NaOH. The amount of 4-hydroquioline formed was determined through spectrofluorimetric readout with the emission detection at 380 nm and excitation wavelength 310 nm.

5. PDE3A & PDE4D2: Enzyme and test compound were preincubated for 15 minutes at room temp before substrate addition. cAMP substrate (at a concentration equal to EC_{80}) was added and incubated at room temperature for 30 minutes. Enzyme reaction was terminated by addition of 9 mM IBMX. Signal was detected using the HitHunter® cAMP detection kit.

Signal Detection

1. For each assay, microplates were transferred to a PerkinElmer Envision™ instrument and readout as described.

Data Analysis

1. Compound activity was analyzed using CBIS data analysis suite (ChemInnovation, CA).

2. For enzyme activity assays, percentage inhibition was calculated using the following formula:
   % Inhibition =100% x (1 - (mean RLU of test sample - mean RLU of vehicle control) / (mean RLU of positive control - mean RLU of vehicle control)).

For Primary screens, percent response was capped at 0% or 100% where calculated percent response returned a negative value or a value greater than 100, respectively.

Supplemental Table S1

Continued on next page.

Supplemental Table S1: IC50 Values for CTP as performed and reported by Eurofins in their Ion Channel Cardiac Profiler Panel.

Estimated IC_{50} Compound Summary Table

Compound	Estimated IC_{50} (μM)						
	Nav1.5 (Peak)	Kv4.3/ KChiP2	Cav1.2	Nav1.5 (Late, Antagonist)	hERG	KCNQ1/min K	Kir2.1
CTP	N/C	N/C	N/C	N/C	N/C	N/C	N/C

*N/C: Not calculable, inhibition >25% was not observed.

REFERENCE COMPOUND RESULTS

CardiacProfiler Reference Compound Panel

ITEM	Assay Name	Mode	Reference Compound	Estimated IC_{50} (μM)
CYL8004QB2DR	hNav1.5 Sodium Channel Assay Qube APC (1 conc.)	Antagonist	Tetracaine	2.8
CYL8069QB2DR	hKv4.3/hKChIP2 Potassium Channel Assay Qube APC (1 conc.)	Antagonist	Flecainide	12.5
CYL8051QB2DR	hCav1.2 (L-type) Calcium Channel Assay Qube APC (1 conc.)	Antagonist	Nifedipine	0.16
CYL7004QB2DR	hNav1.5 Late Current Sodium Channel Assay Qube APC (1 conc.)	Antagonist	Ranolazine	72.5
CYL8038QB2DR	hERG Human Potassium Channel Assay Qube APC (1 conc.)	Antagonist	Verapamil	0.40
CYL8007QB2DR	hKCNQ1/hminK Potassium Channel Assay Qube APC (1 conc.)	Antagonist	Chromanol 293B	21.5
CYL8032QB2DR	hKir2.1 Potassium Channel Assay Qube APC (1 conc.)	Antagonist	Barium Chloride	17.7

Supplemental Figure S1

Continued on next page.

Supplemental Figure S1: Test dose response curves for all 78 assays reported by Eurofins in their SAFETYscan E/IC50 ELECT Service.

Results: Reference Controls

Compound Name	Target Class	Assay Name	Mode	Assay Target	Result Type	RC50 (µM)
NECA	GPCR	Calcium Flux	Agonist	ADORA2A	EC50	0.01669
SCH 442416	GPCR	Calcium Flux	Antagonist	ADORA2A	IC50	0.06547
A 61603 Hydrobromide	GPCR	Calcium Flux	Agonist	ADRA1A	EC50	8e-05
Tamsulosin	GPCR	Calcium Flux	Antagonist	ADRA1A	IC50	0.00094
UK 14304	GPCR	cAMP	Agonist	ADRA2A	EC50	7e-05
Yohimbine	GPCR	cAMP	Antagonist	ADRA2A	IC50	0.00349
(-)-Isoproterenol	GPCR	cAMP	Agonist	ADRB1	EC50	0.00081
Betaxolol	GPCR	cAMP	Antagonist	ADRB1	IC50	0.00361
(-)-Isoproterenol	GPCR	cAMP	Agonist	ADRB2	EC50	0.00069
ICI 118,551 hydrochloride	GPCR	cAMP	Antagonist	ADRB2	IC50	0.00072
[Arg8]-Vasopressin	GPCR	Calcium Flux	Agonist	AVPR1A	EC50	0.00054
SR 49059	GPCR	Calcium Flux	Antagonist	AVPR1A	IC50	0.00112
Sincalide	GPCR	Calcium Flux	Agonist	CCKAR	EC50	6e-05
SR 27897	GPCR	Calcium Flux	Antagonist	CCKAR	IC50	0.03081
Acetylcholine chloride	GPCR	Calcium Flux	Agonist	CHRM1	EC50	0.01552
Atropine	GPCR	Calcium Flux	Antagonist	CHRM1	IC50	0.00342
Acetylcholine chloride	GPCR	cAMP	Agonist	CHRM2	EC50	0.02554
Atropine	GPCR	cAMP	Antagonist	CHRM2	IC50	0.00509
Acetylcholine chloride	GPCR	Calcium Flux	Agonist	CHRM3	EC50	0.06237
Atropine	GPCR	Calcium Flux	Antagonist	CHRM3	IC50	0.0012
CP 55940	GPCR	cAMP	Agonist	CNR1	EC50	1e-05
AM 251	GPCR	cAMP	Antagonist	CNR1	IC50	0.0012
CP 55940	GPCR	cAMP	Agonist	CNR2	EC50	0.00018
SR 144528	GPCR	cAMP	Antagonist	CNR2	IC50	0.06294
Dopamine	GPCR	cAMP	Agonist	DRD1	EC50	0.09828
SCH 39166	GPCR	cAMP	Antagonist	DRD1	IC50	0.00194
Dopamine	GPCR	cAMP	Agonist	DRD2S	EC50	0.00204
Risperidone	GPCR	cAMP	Antagonist	DRD2S	IC50	0.00098
Endothelin 1	GPCR	Calcium Flux	Agonist	EDNRA	EC50	0.00165
BMS 182874	GPCR	Calcium Flux	Antagonist	EDNRA	IC50	0.22412
Histamine	GPCR	Calcium Flux	Agonist	HRH1	EC50	0.00829
Mepyramine	GPCR	Calcium Flux	Antagonist	HRH1	IC50	0.00296
Histamine	GPCR	cAMP	Agonist	HRH2	EC50	0.20972
Tiotidine	GPCR	cAMP	Antagonist	HRH2	IC50	0.13211
Serotonin Hydrochloride	GPCR	cAMP	Agonist	HTR1A	EC50	0.01164
Spiperone	GPCR	cAMP	Antagonist	HTR1A	IC50	0.0586
Serotonin Hydrochloride	GPCR	cAMP	Agonist	HTR1B	EC50	0.00056
SB 224289	GPCR	cAMP	Antagonist	HTR1B	IC50	0.00619
Serotonin Hydrochloride	GPCR	Calcium Flux	Agonist	HTR2A	EC50	0.00165
Altanserin	GPCR	Calcium Flux	Antagonist	HTR2A	IC50	0.01305

Figure 1: Table of control values.

Results: Reference Controls (cont.)

Compound Name	Target Class	Assay Name	Mode	Assay Target	Result Type	RC50 (µM)
Serotonin Hydrochloride	GPCR	Calcium Flux	Agonist	HTR2B	EC50	0.00183
LY 272015	GPCR	Calcium Flux	Antagonist	HTR2B	IC50	0.00063
DADLE	GPCR	cAMP	Agonist	OPRD1	EC50	7e-05
Naltriben	GPCR	cAMP	Antagonist	OPRD1	IC50	0.00053
Dynorphin A (1-17)	GPCR	cAMP	Agonist	OPRK1	EC50	0.06063
nor-Binaltorphimine	GPCR	cAMP	Antagonist	OPRK1	IC50	0.00285
DAMGO	GPCR	cAMP	Agonist	OPRM1	EC50	0.00186
Naloxone	GPCR	cAMP	Antagonist	OPRM1	IC50	0.00493
Isradipine	Ion Channel	Ion Channel	Blocker	CAV1.2	IC50	0.00866
Picrotoxin	Ion Channel	Ion Channel	Blocker	GABAA	IC50	1.82791
GABA	Ion Channel	Ion Channel	Opener	GABAA	EC50	7.6287
Astemizole	Ion Channel	Ion Channel	Blocker	hERG	IC50	0.18015
Bemesetron	Ion Channel	Ion Channel	Blocker	HTR3A	IC50	0.00342
Serotonin Hydrochloride	Ion Channel	Ion Channel	Opener	HTR3A	EC50	0.28391
XE 991	Ion Channel	Ion Channel	Blocker	KvLQT1/minK	IC50	1.05231
ML-277	Ion Channel	Ion Channel	Opener	KvLQT1/minK	EC50	2.49276
Dihydro-ÃŸ-erythroidine	Ion Channel	Ion Channel	Blocker	nAChR(a4/b2)	IC50	0.47546
(-)-Nicotine	Ion Channel	Ion Channel	Opener	nAChR(a4/b2)	EC50	2.09854
Lidocaine Hydrochloride	Ion Channel	Ion Channel	Blocker	NAV1.5	IC50	32.86261
(+)-MK 801	Ion Channel	Ion Channel	Blocker	NMDAR (1A/2B)	IC50	0.07325
L-Glutamic Acid	Ion Channel	Ion Channel	Opener	NMDAR (1A/2B)	EC50	0.38546
BMS-754807	Kinases	Binding	Inhibitor	INSR	IC50	0.00066
Gleevec	Kinases	Binding	Inhibitor	LCK	IC50	0.03045
Staurosporine	Kinases	Binding	Inhibitor	ROCK1	IC50	5e-04
SU-11248	Kinases	Binding	Inhibitor	VEGFR2	IC50	0.00016
BMS-564929	NHR	NHR Nuclear Translocation	Agonist	AR	EC50	0.00161
Geldanamycin	NHR	NHR Nuclear Translocation	Antagonist	AR	IC50	0.06695
Dexamethasone	NHR	NHR Protein Interaction	Agonist	GR	EC50	0.04678
Mifepristone	NHR	NHR Protein Interaction	Antagonist	GR	IC50	0.06916
Physostigmine	Non-Kinase Enzymes	Enzymatic	Inhibitor	AChE	IC50	0.03856
Indomethacin	Non-Kinase Enzymes	Enzymatic	Inhibitor	COX1	IC50	0.03644
NS-398	Non-Kinase Enzymes	Enzymatic	Inhibitor	COX2	IC50	0.19327
Clorgyline	Non-Kinase Enzymes	Enzymatic	Inhibitor	MAOA	IC50	0.00121
Cilostamide	Non-Kinase Enzymes	Enzymatic	Inhibitor	PDE3A	IC50	0.06477
Cilomilast	Non-Kinase Enzymes	Enzymatic	Inhibitor	PDE4D2	IC50	0.01914
GBR 12909	Transporter	Transporter	Blocker	DAT	IC50	0.00123
Desipramine	Transporter	Transporter	Blocker	NET	IC50	0.00837
Clomipramine	Transporter	Transporter	Blocker	SERT	IC50	0.00294

Figure 1 (cont.): Table of control values.

Results: Test Compound(s)

Data shown was normalized to the maximal and minimal response observed in the presence of control ligand and vehicle respectively (y-axis) and is plotted against the corresponding compound concentration in nM in log10 scale (x-axis).

CTP
ADORA2A Agonist
RC50 (uM) > 30

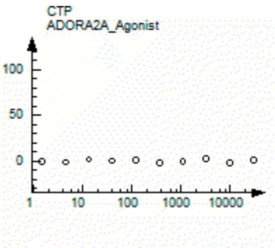

CTP
ADORA2A Antagonist
RC50 (uM) > 30

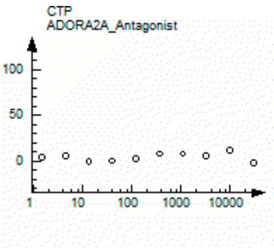

CTP
ADRA1A Agonist
RC50 (uM) > 30

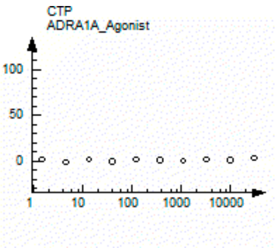

CTP
ADRA1A Antagonist
RC50 (uM) > 30

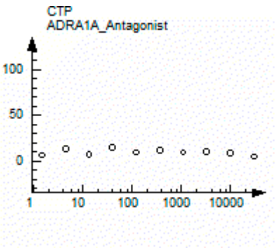

CTP
ADRA2A Agonist
RC50 (uM) > 30

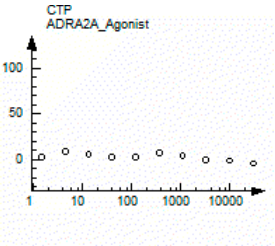

CTP
ADRA2A Antagonist
RC50 (uM) > 30

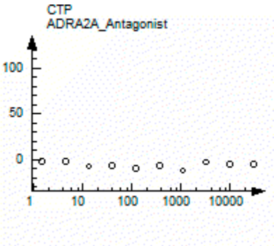

CTP
ADRB1 Agonist
RC50 (uM) > 30

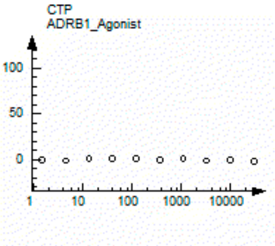

CTP
ADRB1 Antagonist
RC50 (uM) > 30

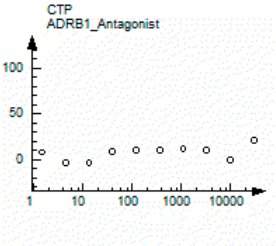

CTP
ADRB2 Agonist
RC50 (uM) > 30

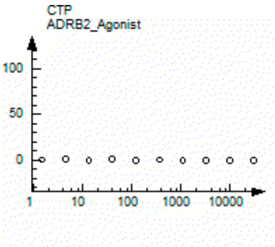

CTP
ADRB2 Agonist
RC50 (uM) > 30

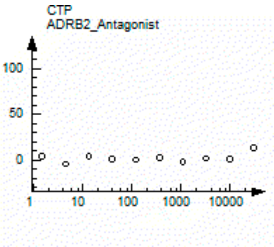

CTP
AVPR1A Agonist
RC50 (uM) > 30

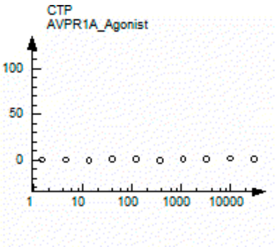

CTP
AVPR1A Antagonist
RC50 (uM) > 30

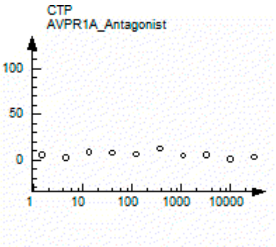

CTP
CCKAR Agonist
RC50 (uM) > 30

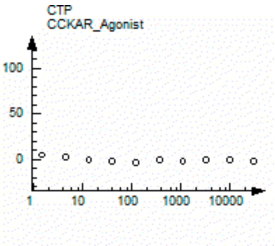

CTP
CCKAR Antagonist
RC50 (uM) > 30

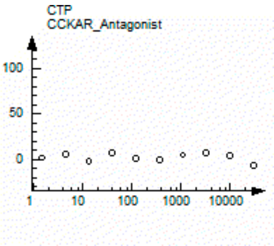

CTP
CHRM1 Agonist
RC50 (uM) > 30

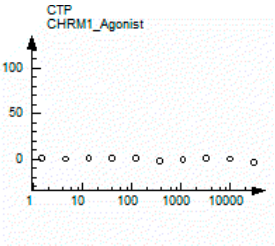

CTP
CHRM1 Antagonist
RC50 (uM) > 30

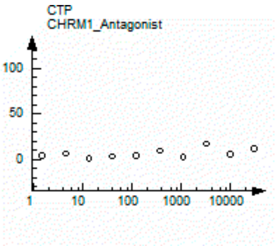

Figure 2: Test dose response curves for 78 assays tested.

CTP
CHRM2 Agonist
RC50 (uM) > 30

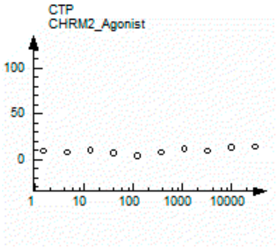

CTP
CHRM2 Antagonist
RC50 (uM) > 30

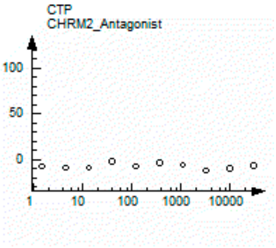

CTP
CHRM3 Agonist
RC50 (uM) > 30

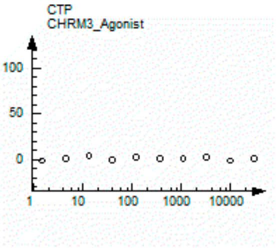

CTP
CHRM3 Antagonist
RC50 (uM) > 30

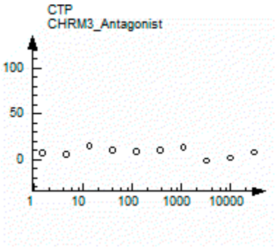

CTP
CNR1 Agonist
RC50 (uM) > 30

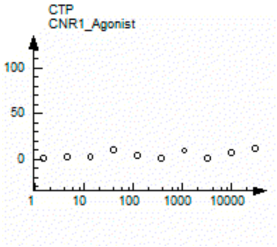

CTP
CNR1 Antagonist
RC50 (uM) > 30

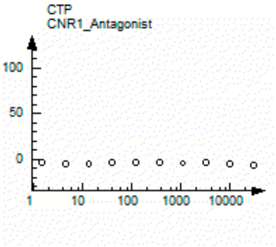

CTP
CNR2 Agonist
RC50 (uM) > 30

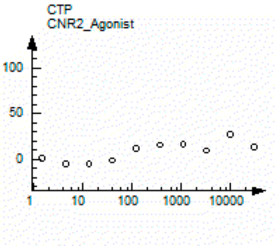

CTP
CNR2 Antagonist
RC50 (uM) > 30

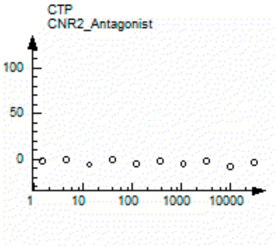

CTP
DRD1 Agonist
RC50 (uM) > 30

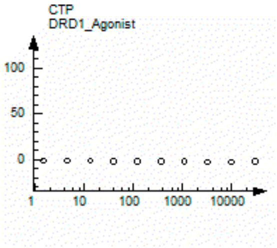

CTP
DRD1 Antagonist
RC50 (uM) > 30

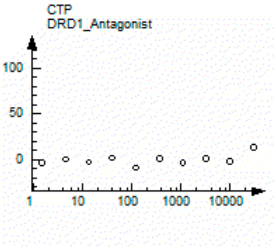

CTP
DRD2S Agonist
RC50 (uM) > 30

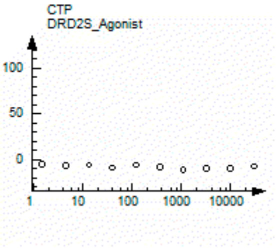

CTP
DRD2S Antagonist
RC50 (uM) > 30

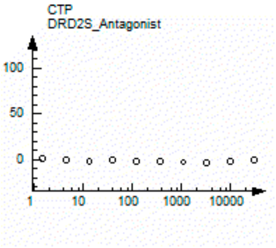

CTP
EDNRA Agonist
RC50 (uM) > 30

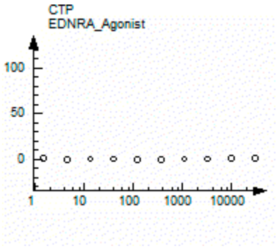

CTP
EDNRA Antagonist
RC50 (uM) > 30

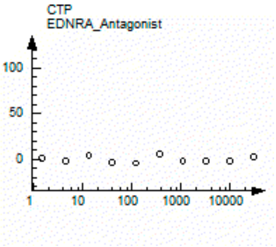

CTP
HRH1 Agonist
RC50 (uM) > 30

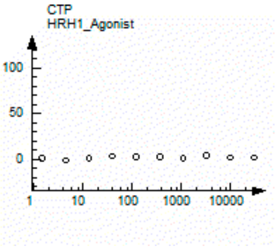

CTP
HRH1 Antagonist
RC50 (uM) > 30

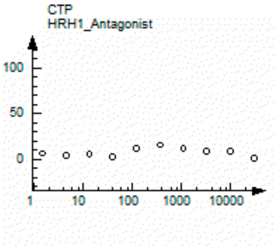

Figure 2 (cont.): Test dose response curves for 78 assays tested.

CTP
HRH2 Agonist
RC50 (uM) > 30

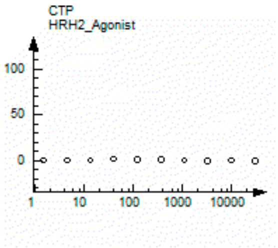

CTP
HRH2 Antagonist
RC50 (uM) > 30

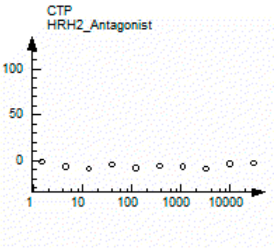

CTP
HTR1A Agonist
RC50 (uM) > 30

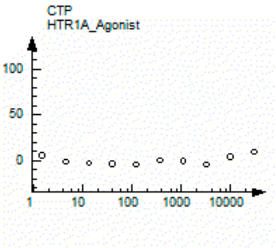

CTP
HTR1A Antagonist
RC50 (uM) > 30

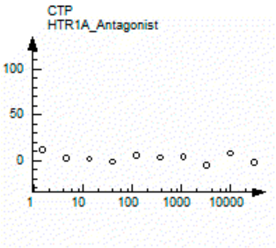

CTP
HTR1B Agonist
RC50 (uM) > 30

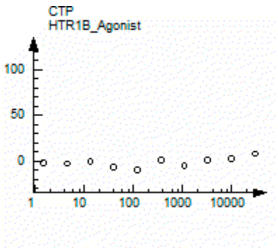

CTP
HTR1B Antagonist
RC50 (uM) > 30

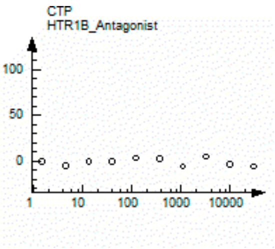

CTP
HTR2A Agonist
RC50 (uM) > 30

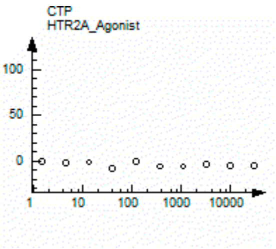

CTP
HTR2A Antagonist
RC50 (uM) > 30

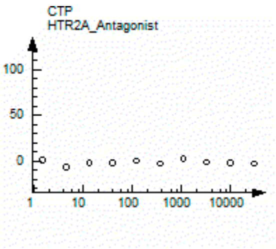

CTP
HTR2B Agonist
RC50 (uM) > 30

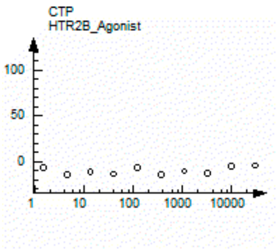

CTP
HTR2B Antagonist
RC50 (uM) > 30

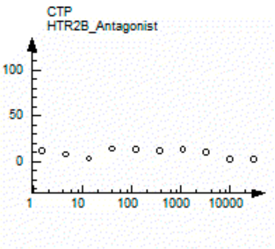

CTP
OPRD1 Agonist
RC50 (uM) > 30

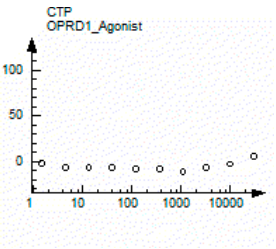

CTP
OPRD1 Antagonist
RC50 (uM) > 30

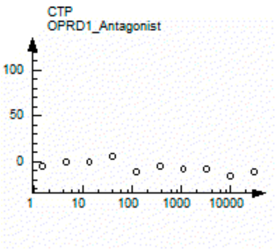

CTP
OPRK1 Agonist
RC50 (uM) > 30

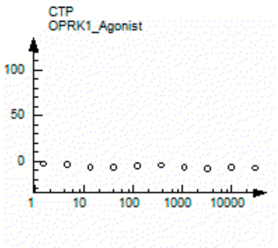

CTP
OPRK1 Antagonist
RC50 (uM) > 30

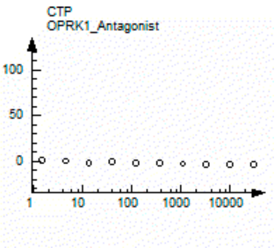

CTP
OPRM1 Agonist
RC50 (uM) = 24.97469

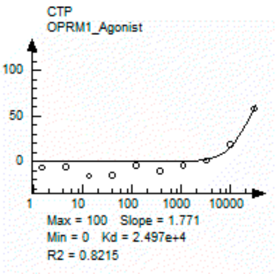

CTP
OPRM1 Antagonist
RC50 (uM) > 30

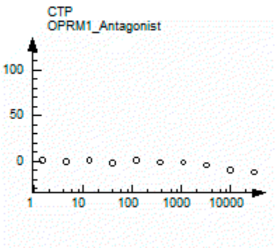

Figure 2 (cont.): Test dose response curves for 78 assays tested.

CTP
CAV1.2
RC50 (uM) > 30

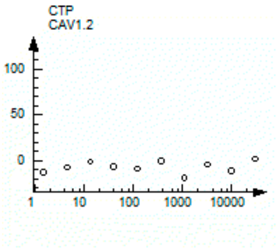

CTP
GABAA Antagonist
RC50 (uM) > 30

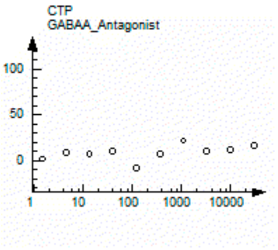

CTP
GABAA Agonist
RC50 (uM) > 30

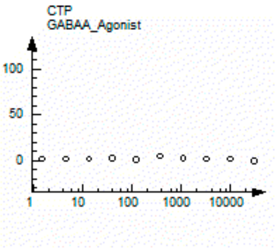

CTP
hERG
RC50 (uM) > 30

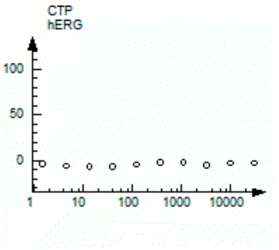

CTP
HTR3A Antagonist
RC50 (uM) > 30

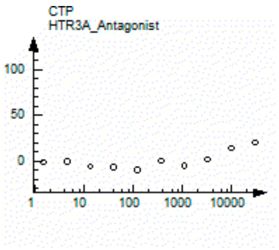

CTP
HTR3A Agonist
RC50 (uM) > 30

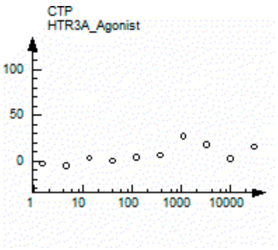

CTP
KvLQT1/minK Antagonist
RC50 (uM) > 30

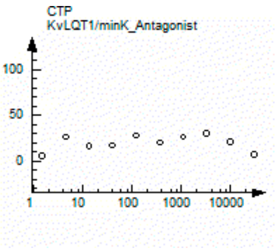

CTP
KvLQT1/minK Agonist
RC50 (uM) > 30

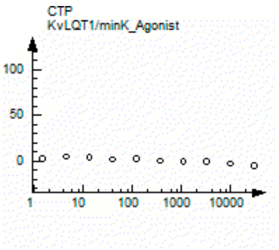

CTP
nAChR(a4/b2) Antagonist
RC50 (uM) > 30

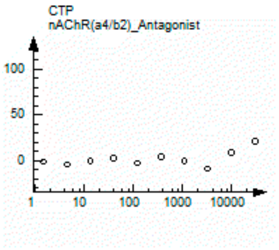

CTP
nAChR(a4/b2) Agonist
RC50 (uM) > 30

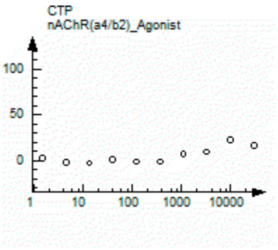

CTP
NAV1.5
RC50 (uM) > 30

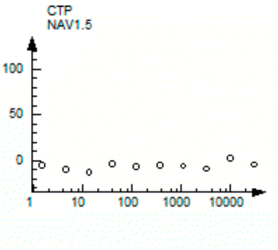

CTP
NMDAR 1A/2B Antagonist
RC50 (uM) > 30

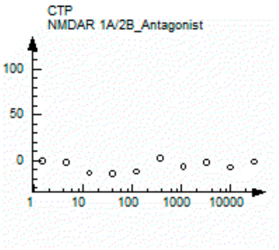

CTP
NMDAR 1A/2B Agonist
RC50 (uM) > 30

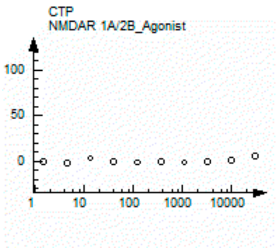

CTP
INSR
RC50 (uM) > 30

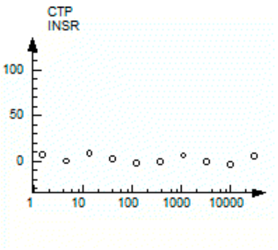

CTP
LCK
RC50 (uM) > 30

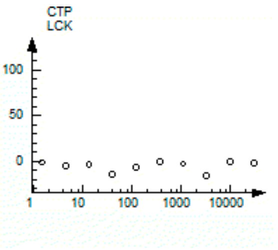

CTP
ROCK1
RC50 (uM) > 30

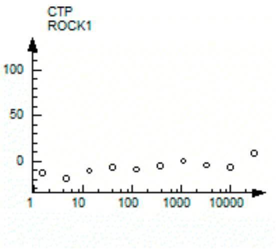

Figure 2 (cont.): Test dose response curves for 78 assays tested.

CTP
VEGFR2
RC50 (uM) > 30

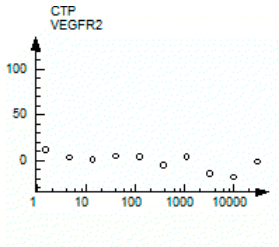

CTP
AR Agonist
RC50 (uM) > 30

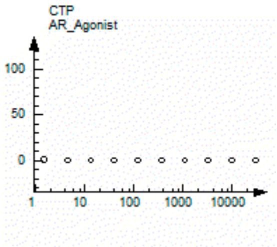

CTP
AR Antagonist
RC50 (uM) > 30

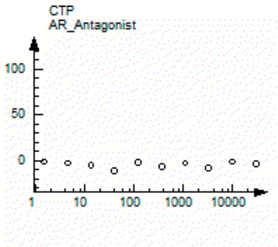

CTP
GR Agonist
RC50 (uM) > 30

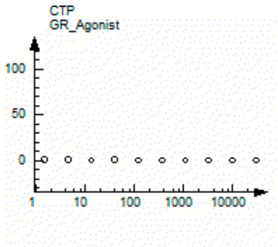

CTP
GR Antagonist
RC50 (uM) > 30

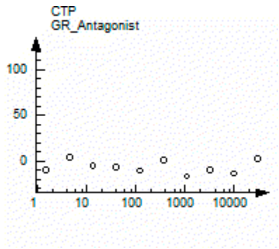

CTP
AChE
RC50 (uM) > 30

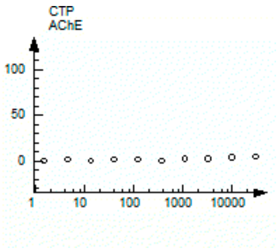

CTP
COX1
RC50 (uM) > 30

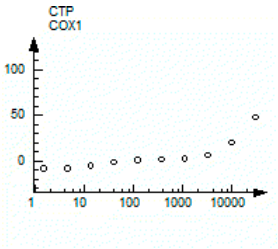

CTP
COX2
RC50 (uM) = 16.86902

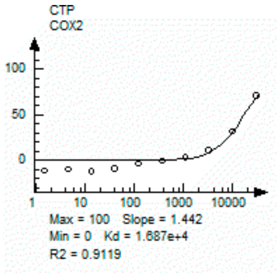

CTP
MAOA
RC50 (uM) > 30

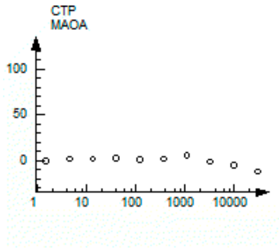

CTP
PDE3A
RC50 (uM) > 30

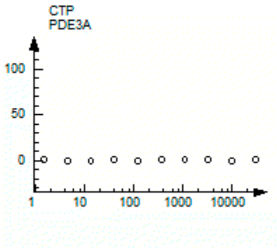

CTP
PDE4D2
RC50 (uM) > 30

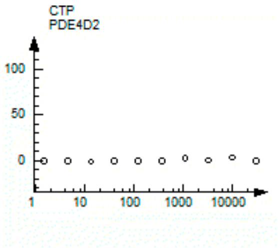

CTP
DAT
RC50 (uM) > 30

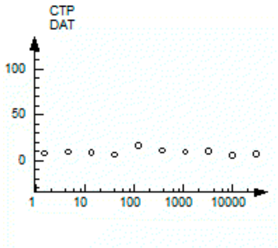

CTP
NET
RC50 (uM) > 30

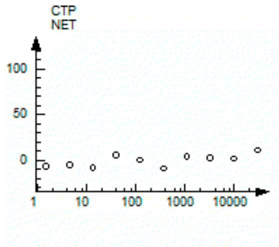

CTP
SERT
RC50 (uM) > 30

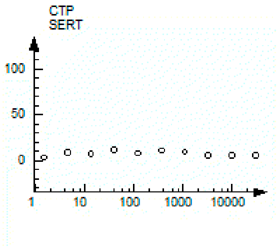

Figure 2 (cont.): Test dose response curves for 78 assays tested.

Results: Summary Table

Compound Name	Order ID	Target Class	Assay Name	Mode	Assay Target	Result Type	Value Prefix	RC50 (µM)	Hill	Curve Bottom	Curve Top	Max Response
CTP	US073-0027327-O	GPCR	Calcium Flux	Agonist	ADORA2A	EC50	>	30				0.15
CTP	US073-0027327-O	GPCR	Calcium Flux	Agonist	ADRA1A	EC50	>	30				2.35
CTP	US073-0027327-O	GPCR	Calcium Flux	Agonist	AVPR1A	EC50	>	30				0.8
CTP	US073-0027327-O	GPCR	Calcium Flux	Agonist	CCKAR	EC50	>	30				0
CTP	US073-0027327-O	GPCR	Calcium Flux	Agonist	CHRM1	EC50	>	30				0
CTP	US073-0027327-O	GPCR	Calcium Flux	Agonist	CHRM3	EC50	>	30				0
CTP	US073-0027327-O	GPCR	Calcium Flux	Agonist	EDNRA	EC50	>	30				0.24
CTP	US073-0027327-O	GPCR	Calcium Flux	Agonist	HRH1	EC50	>	30				1.06
CTP	US073-0027327-O	GPCR	Calcium Flux	Agonist	HTR2A	EC50	>	30				0
CTP	US073-0027327-O	GPCR	Calcium Flux	Agonist	HTR2B	EC50	>	30				0
CTP	US073-0027327-O	GPCR	Calcium Flux	Antagonist	ADORA2A	IC50	>	30				11.4
CTP	US073-0027327-O	GPCR	Calcium Flux	Antagonist	ADRA1A	IC50	>	30				8.01
CTP	US073-0027327-O	GPCR	Calcium Flux	Antagonist	AVPR1A	IC50	>	30				2.67
CTP	US073-0027327-O	GPCR	Calcium Flux	Antagonist	CCKAR	IC50	>	30				3.63
CTP	US073-0027327-O	GPCR	Calcium Flux	Antagonist	CHRM1	IC50	>	30				11.26
CTP	US073-0027327-O	GPCR	Calcium Flux	Antagonist	CHRM3	IC50	>	30				7.21
CTP	US073-0027327-O	GPCR	Calcium Flux	Antagonist	EDNRA	IC50	>	30				1.61
CTP	US073-0027327-O	GPCR	Calcium Flux	Antagonist	HRH1	IC50	>	30				8.02
CTP	US073-0027327-O	GPCR	Calcium Flux	Antagonist	HTR2A	IC50	>	30				0
CTP	US073-0027327-O	GPCR	Calcium Flux	Antagonist	HTR2B	IC50	>	30				2.26
CTP	US073-0027327-O	GPCR	cAMP	Agonist	ADRA2A	EC50	>	30				0
CTP	US073-0027327-O	GPCR	cAMP	Agonist	ADRB1	EC50	>	30				0
CTP	US073-0027327-O	GPCR	cAMP	Agonist	ADRB2	EC50	>	30				0
CTP	US073-0027327-O	GPCR	cAMP	Agonist	CHRM2	EC50	>	30				13.56
CTP	US073-0027327-O	GPCR	cAMP	Agonist	CNR1	EC50	>	30				10.79
CTP	US073-0027327-O	GPCR	cAMP	Agonist	CNR2	EC50	>	30				26.46

Figure 3: Summary of compound(s) tested

Results: Summary Table (cont.)

Compound Name	Order ID	Target Class	Assay Name	Mode	Assay Target	Result Type	Value Prefix	RC50 (μM)	Hill	Curve Bottom	Curve Top	Max Response
CTP	US073-0027327-O	GPCR	cAMP	Agonist	DRD1	EC50	>	30				0
CTP	US073-0027327-O	GPCR	cAMP	Agonist	DRD2S	EC50	>	30				0
CTP	US073-0027327-O	GPCR	cAMP	Agonist	HRH2	EC50	>	30				0
CTP	US073-0027327-O	GPCR	cAMP	Agonist	HTR1A	EC50	>	30				8.71
CTP	US073-0027327-O	GPCR	cAMP	Agonist	HTR1B	EC50	>	30				6.87
CTP	US073-0027327-O	GPCR	cAMP	Agonist	OPRD1	EC50	>	30				5.23
CTP	US073-0027327-O	GPCR	cAMP	Agonist	OPRK1	EC50	>	30				0
CTP	US073-0027327-O	GPCR	cAMP	Agonist	OPRM1	EC50	=	24.97469	1.77	0	100	57.51
CTP	US073-0027327-O	GPCR	cAMP	Antagonist	ADRA2A	IC50	>	30				0
CTP	US073-0027327-O	GPCR	cAMP	Antagonist	ADRB1	IC50	>	30				20.12
CTP	US073-0027327-O	GPCR	cAMP	Antagonist	ADRB2	IC50	>	30				12.08
CTP	US073-0027327-O	GPCR	cAMP	Antagonist	CHRM2	IC50	>	30				0
CTP	US073-0027327-O	GPCR	cAMP	Antagonist	CNR1	IC50	>	30				0
CTP	US073-0027327-O	GPCR	cAMP	Antagonist	CNR2	IC50	>	30				0
CTP	US073-0027327-O	GPCR	cAMP	Antagonist	DRD1	IC50	>	30				12.88
CTP	US073-0027327-O	GPCR	cAMP	Antagonist	DRD2S	IC50	>	30				0
CTP	US073-0027327-O	GPCR	cAMP	Antagonist	HRH2	IC50	>	30				0
CTP	US073-0027327-O	GPCR	cAMP	Antagonist	HTR1A	IC50	>	30				7.24
CTP	US073-0027327-O	GPCR	cAMP	Antagonist	HTR1B	IC50	>	30				0
CTP	US073-0027327-O	GPCR	cAMP	Antagonist	OPRD1	IC50	>	30				0
CTP	US073-0027327-O	GPCR	cAMP	Antagonist	OPRK1	IC50	>	30				0
CTP	US073-0027327-O	GPCR	cAMP	Antagonist	OPRM1	IC50	>	30				0
CTP	US073-0027327-O	Ion Channel	Ion Channel	Blocker	CAV1.2	IC50	>	30				0.86
CTP	US073-0027327-O	Ion Channel	Ion Channel	Blocker	GABAA	IC50	>	30				15.25
CTP	US073-0027327-O	Ion Channel	Ion Channel	Blocker	hERG	IC50	>	30				0
CTP	US073-0027327-O	Ion Channel	Ion Channel	Blocker	HTR3A	IC50	>	30				19.22

Figure 3 (cont.): Summary of compound(s) tested

Results: Summary Table (cont.)

Compound Name	Order ID	Target Class	Assay Name	Mode	Assay Target	Result Type	Value Prefix	RC50 (µM)	Hill	Curve Bottom	Curve Top	Max Response
CTP	US073-0027327-O	Ion Channel	Ion Channel	Blocker	KvLQT1/minK	IC50	>	30				19.89
CTP	US073-0027327-O	Ion Channel	Ion Channel	Blocker	nAChR(a4/b2)	IC50	>	30				20.39
CTP	US073-0027327-O	Ion Channel	Ion Channel	Blocker	NAV1.5	IC50	>	30				1.75
CTP	US073-0027327-O	Ion Channel	Ion Channel	Blocker	NMDAR (1A/2B)	IC50	>	30				0
CTP	US073-0027327-O	Ion Channel	Ion Channel	Opener	GABAA	EC50	>	30				1.09
CTP	US073-0027327-O	Ion Channel	Ion Channel	Opener	HTR3A	EC50	>	30				14.86
CTP	US073-0027327-O	Ion Channel	Ion Channel	Opener	KvLQT1/minK	EC50	>	30				0
CTP	US073-0027327-O	Ion Channel	Ion Channel	Opener	nAChR(a4/b2)	EC50	>	30				21.44
CTP	US073-0027327-O	Ion Channel	Ion Channel	Opener	NMDAR (1A/2B)	EC50	>	30				4.77
CTP	US073-0027327-O	Kinases	Binding	Inhibitor	INSR	IC50	>	30				4.98
CTP	US073-0027327-O	Kinases	Binding	Inhibitor	LCK	IC50	>	30				0
CTP	US073-0027327-O	Kinases	Binding	Inhibitor	ROCK1	IC50	>	30				7.43
CTP	US073-0027327-O	Kinases	Binding	Inhibitor	VEGFR2	IC50	>	30				0
CTP	US073-0027327-O	NHR	NHR Nuclear Translocation	Agonist	AR	EC50	>	30				0
CTP	US073-0027327-O	NHR	NHR Nuclear Translocation	Antagonist	AR	IC50	>	30				0
CTP	US073-0027327-O	NHR	NHR Protein Interaction	Agonist	GR	EC50	>	30				0
CTP	US073-0027327-O	NHR	NHR Protein Interaction	Antagonist	GR	IC50	>	30				2.14
CTP	US073-0027327-O	Non-Kinase Enzymes	Enzymatic	Inhibitor	AChE	IC50	>	30				3.74
CTP	US073-0027327-O	Non-Kinase Enzymes	Enzymatic	Inhibitor	COX1	IC50	>	30				47
CTP	US073-0027327-O	Non-Kinase Enzymes	Enzymatic	Inhibitor	COX2	IC50	=	16.86902	1.44	0	100	70.14
CTP	US073-0027327-O	Non-Kinase Enzymes	Enzymatic	Inhibitor	MAOA	IC50	>	30				0
CTP	US073-0027327-O	Non-Kinase Enzymes	Enzymatic	Inhibitor	PDE3A	IC50	>	30				0
CTP	US073-0027327-O	Non-Kinase Enzymes	Enzymatic	Inhibitor	PDE4D2	IC50	>	30				2.3
CTP	US073-0027327-O	Transporter	Transporter	Blocker	DAT	IC50	>	30				6
CTP	US073-0027327-O	Transporter	Transporter	Blocker	NET	IC50	>	30				9.99
CTP	US073-0027327-O	Transporter	Transporter	Blocker	SERT	IC50	>	30				4.74

Figure 3 (cont.): Summary of compound(s) tested
